# Consumption of Nutrition Supplements Is Associated with Less Hypoglycemia during Admission—Results from the MENU Project

**DOI:** 10.3390/nu11081832

**Published:** 2019-08-08

**Authors:** Eyal Leibovitz, Fariba Moore, Irina Mintser, Anat Levi, Ron Dubinsky, Mona Boaz

**Affiliations:** 1 Department of Internal Medicine “A”, Yoseftal Hospital, Yotam Road, Eilat 88000, Israel; 2 Departments of Internal Medicine “E”, Wolfson Medical Center, Holon 58100, Israel; 3 Departments of Internal Medicine “F”, Wolfson Medical Center, Holon 58100, Israel; 4 Department of Nutrition, Wolfson Medical Center, Holon 58100, Israel; 5 Department of Nutrition Sciences, Ariel University, Ariel 40700, Israel

**Keywords:** albumin, diabetes mellitus, hypoglycemia, oral nutrition supplement

## Abstract

Aim: We studied the effect of the addition of an oral nutrition supplement (ONS) on the rate of hypoglyemia among hospitalized type 2 diabetes mellitus (DM) patients. Methods: In this retrospective analysis, all DM patients with hypoalbuminemia (albumin < 3.5 g/dL) admitted to internal medicine “E” at Wolfson Medical Center between 1 June 2016 and 30 April 2017 were included. One bottle of ONS (Glucerna, 330 KCAL, 28 g carbohydrates, 17 g protein, 17 g fat) was added to the morning meal. The consumption of the ONS was verified during the morning rounds. All glucose measurements were recorded automatically in the patients’ electronic medical records. A logistic regression model was used to evaluate the effect of the nutrition support on the occurrence of hypoglycemia. Results: 218 patients (mean age 77.4 ± 12.0 years, 63.3% female, mean albumin 3.13 ± 0.32 g/dL), of whom 27.9% had documented hypoglycemia during hospitalization were included. The patients consumed 69.5% ± 37.1 of the ONS provided, and ONS was started 4.3 ± 5.3 days from admission. A logistic regression model indicated that age (Odds ratio [OR] 1.048, 95% CI 1.014–1.083, *p* = 0.005), insulin treatment (OR 3.059, 95% CI 1.497–6.251, *p* = 0.002), and the day of ONS started from admission (OR 1.094, 95% CI 1.021–1.173, *p* = 0.011) were associated with an increased risk of hypoglycemia. Complete consumption of the ONS was associated with a reduced risk of hypoglycemia: OR 0.364, 95% CI 0.149–0.890, *p* = 0.027. Age, other DM medications and serum albumin did not affect the risk. Conclusion: The intake of a complete serving of ONS may be associated with a reduction of the risk of hypoglycemia among diabetes in-patients with hypoalbuminemia.

## 1. Background

Glucose control, even for the short duration of the hospital stay, significantly affects the length of hospital stay and mortality, regardless of the reason for admission [1]. A key factor associated with poor outcome is hypoglycemia, which has been shown to independently increase morbidity and hospital mortality [2]. Hypoglycemia incidence has been associated with strict glucose control among intensive care units (ICU) [3] as well as non-critically ill patients [4].

The rate of malnutrition among hospitalized individuals is high and, depending on the assessment method used, between 30% and 50% of patients are malnourished or at risk for malnutrition [5]. Recently, we showed an association between nutrition status at hospital admission and hypoglycemia incidence during the hospitalization period. Both malnutrition risk, as measured by the NRS2002 [6] and admission serum albumin [7] were found to be predictive of hypoglycemia. While the NRS2002 was a strong predictor for patients without DM, serum albumin was found to be a stronger predictor of hypoglycemia among DM patients. 

The fact that patients admitted to the hospital consume less than 50% of the food served in hospital [8] contributes to the continuous deterioration of nutrition status [9]. The addition of ONS to polymorbid patients has been shown to be beneficial in some patient populations [10,11]. 

The hypothesis of this study was that hypoglycemia, occurring in hospitalized people with diabetes, represents poor nutritional status; further, hypoglycemia may be prevented with ONS. The purpose of this study was to evaluate the effect of an ONS-based nutrition care program on the hypoglycemia incidence rate among people with diabetes who received ONS. 

## 2. Methods

General description: The Measuring Nutrition In hospitalized patients (MENU) project is a hospital-wide endeavor to study the effect of malnutrition on the general population admitted [5]. The project calls for evaluating malnutrition risk and specific laboratory information and enhance nutrition treatment in specific cases. All patients admitted to internal medicine, surgical and orthopedic units at the Wolfson Medical Center undergo screening for malnutrition using the Nutrition Risk Score (NRS) 2002 or Malnutrition Universal Screening Tool (MUST) score. In addition, patients’ serum albumin levels are measured routinely on the first morning of the week as part of the Sequential Multiple Analysis—Computer (SMAC)16 blood chemistry panel. For cases that test positive for malnutrition screening, and/or for cases with albumin level less than 3.5 g/dL, a request for a dietitian consultation is made. Oral nutrition supplements (ONS) are automatically prescribed for patients at risk of malnutrition as part of the treatment and before the dietician consultation. The dietician in charge can either keep the recommendation in place or change it depending on the additional factors. For cases receiving the ONS, the pack is dispensed with the morning medication (at 8:00 a.m.) and the amount of ONS consumed is estimated at 10:00–11:00 during the morning rounds. For cases that do not receive the daily ONS, the reason is recorded (patient’s refusal, beginning of tube feeding, decision of the medical staff or change in dietician recommendations). 

*Informed consent*: The current study was considered an observational study by the local Helsinky committee, which precluded the need for informed consent.

*Study population*: For the purpose of this study, only patients with type 2 DM and admission serum albumin level below 3.5 g/dL (hypoalbuminemia) were treated with ONS: one bottle of Glucerna: 330 KCAL, 28 g carbohydrates, 17 g protein, 17g fat. 

*Data acquisition period*: Between 1 June 2016 and 30 April 2017.

*Data collection*: Data collected from the patients’ medical records included demographic data (age and sex), reason for admission (either “infection” or “other”), co-morbidities, laboratory data (serum albumin, Hemoglobin, CRP, Creatinine and all glucose measurements), diabetes medications, length of hospital stay and date of death (when applicable). At the Wolfson medical center, all glucose measurements are recorded using an institutional blood glucose monitoring system, which consists of a point-of-care, an automated glucometer and an interactive database [12]. A patient was considered hypoglycemic if he had at least one documented glucose level ≤ 70 mg/dL, as measured by either the glucometer system or the lab chemistry panel, regardless of symptoms. In addition, the daily estimated amount of the ONS consumed and the reasons for lack of consumption/dispensing were recorded. 

For each patient, two variables were created. The first variable was “Day of inclusion” which was calculated as the number of days that passed from the day of hospitalization to the first day the patient started receiving the nutrition supplements. The second variable was “Percent ONS completed from inclusion”, which was calculated as the number of days the patient consumed more than 50% of the ONS dispensed, divided by the number of the days the patient was prescribed the ONS. 

*Statistical Methods*: All data was collected on p paper documents and uploaded to Excel worksheets (v. 2010, Microsoft, Redmond, WA, USA). SPSS v. 24.0 (IBM Inc., New York, NY, USA) was used for all statistical analyses. The distributions of continuous variables were assessed for normality using the Kolmogorov-Smirnov test. The proportion of malnourished individuals was estimated by dividing this number by the total number of newly hospitalized patients surveyed. Continuous variables were described using mean ± standard deviation. Categorical variables, such as malnutrition, were described using frequency counts and expressed as *n* (%). Continuous variables were compared by malnutrition risk and hypoglycemia incidence using the *t*-test for independent samples or the Mann–Whitney U as appropriate. Associations between categorical variables were assessed using the chi square test. To ascertain the effect of malnutrition on hypoglycemia, a logistic regression model was used where hypoglycemia occurrence was the dependent variable, and age, sex and malnutrition status were entered as independent variables. Other models included the same variables, as well as diabetes mellitus status as hypoalbuminemia as covariates. General linear modeling was used to predict the length of hospital stay. Cox regression analysis was used predict 30-day and 6-month mortality. All tests were two-tailed and considered significant if *p* < 0.05.

## 3. Results

### 3.1. General Description of Patient Population

Of the 232 patients who fulfilled the inclusion criteria, 14 (6.0%) were dropped from the analysis because of incomplete records. Thus, 218 patients were included in the analysis: mean age of 77.4 ± 12.0 years, 63.3% female. Mean albumin levels were 3.13 ± 0.32 g/dL and 27.1% of the study population had at least one documented hypoglycemic event during the hospitalization period. 

Table 1 shows the patient characteristics compared across hypoglycemia groups. Diabetes patients with hypoglycemia had higher rates of acute infection as the reason for admission and a tendency towards higher rates of chronic ischemic heart disease and malignancy as co-morbidities. In addition, hypoglycemic patients had lower albumin levels, lower rates of albumin increase during admission and lower hemoglobin compared to non-hypoglycemic diabetes patients. No other laboratory difference was noted, including the first glucose measurement during admission and the average glucose throughout the hospitalization.

Glucose lowering medications were prescribed to 150 (68.8%) patients, and 84 patients (38.5%) received insulin. When analyzing patients according to DM treatment, insulin-treated patients were younger (73.6 ± 12.3 vs. 79.8 ± 11.2, *p* < 0.001), but had a similar length of hospital stay (15.3 ± 13.2 vs. 12.5 ± 12.9 days, *p* = 0.122), a similar duration to the day of inclusion (4.6 ± 5.2 vs. 4.2 ± 5.4 days, *p* = 0.550) and a similar percentage of ONS completion (69.7 ± 38.2 vs. 69.4 ± 36.5%, *p* = 0.951). Similar results were obtained when analyzing patients according to other (non-insulin) DM treatments.

### 3.2. Effect of Nutrition on Hypoglycemia

The average rate of the ONS protocol completion was 69.5% ± 37.1, and the average day of inclusion to the protocol was 4.3 ± 5.3 days (median 3 days, Q1–Q3 2–4 days). 

**Figure 1:**
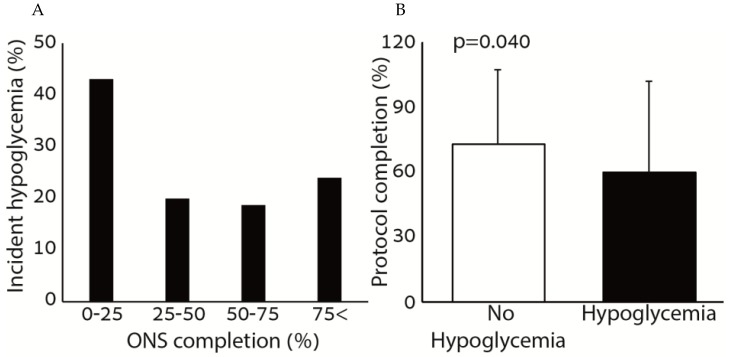
Rate of hypoglycemia according to ONS consumption (**A**) and percentage of ONS protocol completion across hypoglycemia sub-groups (**B**).

Figure 1 shows the association between hypoglycemia and the consumption of ONS. Patients who consumed small amounts of the ONS (0–25%) had higher rates of incident hypoglycemia compared to patients who consumed more than 25% of the ONS prescribed. The percentage of ONS consumed was significantly higher among patients who did not have incident hypoglycemia during the hospitalization period. There was no correlation between the amount of ONS consumed and the average glucose during the hospitalization (r = 0.005, *p* = 0.940).

**Figure 2:**
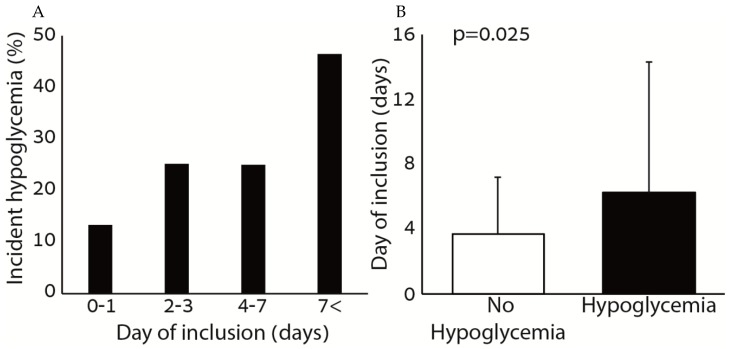
Rate of hypoglycemia according to day of inclusion (**A**) and day of inclusion (**B**) across hypoglycemia groups.

Figure 2 shows the association between hypoglycemia and the day of inclusion to the protocol. There was a positive association between the rate of incident hypoglycemia and the day of inclusion to the protocol. Among patients with hypoglycemia, the day of inclusion was significantly greater compared to patients without hypoglycemia.

To study the impact of the nutrition care program on hypoglycemia occurrence, we used a logistic regression model using hypoglycemia occurrence (“hypoglycemic patients”) as the dependent variable, and age, sex, baseline albumin level, insulin treatment (yes/no), other glucose lowering medications (yes/no) and the percent of ONS completed from inclusion, as well as the inclusion day as covariates. The model showed that age (OR 1.048, 95% confidence interval 1.014–1.083, *p* = 0.005), insulin treatment (OR 3.059, 95% confidence interval 1.497–6.251, *p* = 0.002) and day of inclusion (OR 1.094, 95% confidence interval 1.021–1.173, *p* = 0.011) were associated with an increased risk of hypoglycemia while the percentage of ONS completed from inclusion was associated with a reduction of the risk of hypoglycemia (OR 0.364, 95% confidence interval 0.149–0.890, *p* = 0.027). Sex, albumin level and other glucose lowering medications did not affect the results. No association between the change in albumin level throughout the admission and the percentage of ONS consumed from inclusion (r = −0.043, *p* = 0.526), or the number of days ONS treatment was prescribed (r = −0.100, *p* = 0.142) was noted. 

### 3.3. Effect of Nutrition Intervention According to Sex

There were 138 females and 80 males in the study. Females were older (78.9 ± 10.2 vs. 74.9 ± 14.4, *p* = 0.031), but no difference was noted with regards to admission serum albumin (3.1 ± 0.3 vs. 3.1 ± 0.4, *p* = 0.284), consumption of the ONS (68.2 ± 38.2 vs. 71.8 ± 35.3, *p* = 0.481) and day of inclusion (3.8 ± 3.6 vs. 5.2 ± 7.4, *p* = 0.112). The rate of hypoglycemia was also similar (24.6% vs. 31.3%, *p* = 0.290).

### 3.4. Association of Nutrition Care Program and Hypoglycemia with Prognosis

Patients with hypoglycemia had a prolonged duration of hospital stay compared to non-hypoglycemic patients (19.9 ± 18.0 compared to 11.3 ± 9.8 days, *p* < 0.001). No difference was noted regarding in-hospital mortality rates (2.3% for hypoglycemic patients compared to 0.8% for non-hypoglycemic patients, *p* = 0.461); however, 30-day and 6-month mortality rates were significantly higher among hypoglycemic patients (25.4% and 52.5% vs. 8.2% and 22.6% for the non-hypoglycemic population, *p* = 0.001 and *p* = 0.002 respectively).

**Figure 3:**
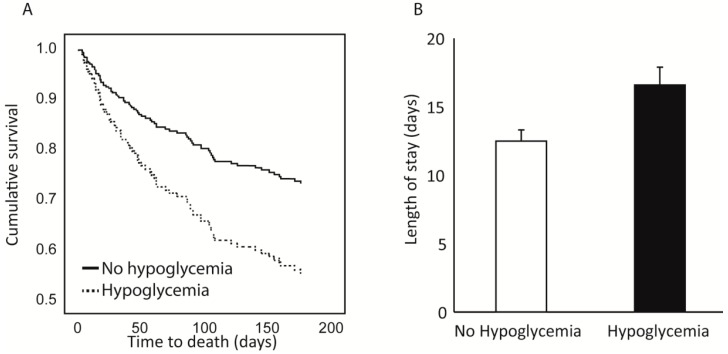
6-month mortality (**A**) and length of hospital stay (**B**) across hypoglycemia groups.

To study the association between the completion of the nutrition care program and the length of the hospital stay, we used a general linear model using length of stay as the dependent variable, hypoglycemia as a fixed factor and age, sex, albumin, albumin increase status, day of inclusion and percentage of ONS completion from inclusion as co-variates. The model showed that hypoglycemia significantly affected the length of stay even after controlling for the other parameters. The estimated marginal means for length of stay were 16.7 ± 1.4 for patients with hypoglycemia compared to 12.6 ± 0.8 for patients without hypoglycemia (Figure 3). The percentage of ONS completion from inclusion also significantly affected the length of stay, but albumin level and albumin increase during the admission did not. 

A cox regression model was used to study the association of the nutrition care program and hypoglycemia with 30-day and 6-month mortality. The co-variates were age, sex, day of inclusion, albumin level, albumin increase status, percentage of ONS completed from inclusion and hypoglycemia occurrence. The model showed that age (HR 1.061, 95% confidence interval 1.014–1.111, *p* = 0.011) and hypoglycemia occurrence (HR 2.764, 95% confidence interval 1.257–6.076, *p* = 0.011) were associated with an increased 30-day mortality, while the percentage of ONS completed from inclusion was associated with a reduced 30-day morality (HR 0.301, 95% confidence interval 0.112–0.805, *p* = 0.017). The day of inclusion, baseline albumin level, and increased albumin during the admission were not associated with a change in 30-day mortality (Figure 3). For 6-month mortality, age (HR 1.057, 95% confidence interval 1.031–1.083, *p* < 0.001), male sex (HR 2.206, 95% confidence interval 1.391–3.498, *p* = 0.001) and hypoglycemia occurrence (HR 1.984, 95% confidence interval 1.226–3.210, *p* = 0.005) were associated with an increased 6-month mortality, while the percentage of ONS completed from inclusion (HR 0.503, 95% confidence interval 0.276–0.915, *p* = 0.024) and baseline albumin level (HR 0.342, 95% confidence interval 0.178–0.659, *p* = 0.001) were associated with a reduced 6-month mortality. The day of inclusion and albumin increase status during the admission did not affect the 6-month mortality rates.

## 4. Discussion

In the present study, two key modifiable elements were associated with the rate of hypoglycemia among diabetes patients. The rate of ONS consumption throughout the admission was associated with a significant (>60%) reduction of hypoglycemia. Additionally, the number of days without nutritional support (described here as “day of inclusion”), was associated with an increase in the rate of hypoglycemia (10% risk increase for each day without ONS). These factors strengthen the association between malnutrition and poor dietary intake with an increased risk of hypoglycemia. 

Our results should encourage the medical staff to adhere to a nutrition care plan for every diabetes patient at risk for developing hypoglycemia during hospitalization, and not to focus exclusively on glucose-lowering medications. The day of inclusion increased risk of hypoglycemia; thus, a nutrition care plan should be implemented as soon as the patient is admitted. Furthermore, the protective effect of ONS on hypoglycemia, 30-day and 6-month mortality underlines the need to encourage ONS consumption and not merely prescribe ONS. 

The association of malnutrition and hypoglycemia^5^ may indicate that malnourished individuals are less able to prevent hypoglycemia occurrence. A reduced food consumption and/or weight loss during the period preceding hospitalization was a significant factor^5^, perhaps implicating reduced glycogen storage as an explanatory mechanism. A reduced food intake during hospitalization, as documented previously, contributes to the deterioration of the nutrition status, and has deleterious repercussions [8]. While nutrition support to prevent hypoglycemia seems logical, ONS-associated outcomes in internal medicine departments have not been reported. 

The mechanisms through which ONS exerts its beneficial effect may include a shift towards liver glycogen formation, counteracting the effects of weight reduction/decreased food intake that may have occurred prior to hospitalization. Additionally, an increased protein intake has been shown to enhance muscle function and possibly improve aerobic exercise capacity [13]. It is possible that the consumption of protein-rich ONS caused a shift of muscle metabolism towards aerobic metabolism and a reduction in the rate of the glucose consumption in the muscle. These possibilities need to be evaluated in other studies. 

We failed to document an association between the rate of ONS consumption and days of treatment with change in albumin level throughout the admission. There are several explanations for this. The first is the fact that we only used one daily dose of ONS for a relatively short period of time. This may be not sufficient to have a significant impact on albumin levels [14]. In addition, albumin is a negative acute phase protein and its levels may indicate other conditions not associated with nutrition status [15]. For this question, pre-albumin may be a better marker [16]. These issues should be investigated in another trial with a longer duration of treatment. 

We documented an extremely high hypoglycemia rate (~27%) in the patient population studied. This was higher than anticipated in reference to previous studies (<10%) In a previous study, we documented a strong association between hypoalbuminemia and incident hypoglycemia [7], and the present patient population had very low albumin levels. This may explain our results. 

The results of the present study must be understood in the framework of design limitations. First, this study is a retrospective analysis of medical charts. We cannot conclude with certainty that the reduced rate of hypoglycemia observed was definitely caused by nutrition intervention. It is possible that this association was confounded by disease severity, such that sicker patients may be more likely to have reduced appetite and thus, consume less food and ONS. 

A major study drawback was that food intake was not measured. Indeed, it is possible for consumption of ONS to have a significant effect on the total caloric intake of the patients. However, it was been previously that ONS is more efficacious in increasing patient caloric intake than protein bars or meals [17], and that the consumption of ONS does not affect the amount of nutrients consumed in hospital meals [18]. Moreover, given the fact that every day without ONS was significantly associated with an increased incidence of hypoglycemia, it is likely that patients did not consume many calories during the period without the ONS and that the addition of ONS had a significant effect on caloric intake. 

We therefore conclude that ONS supplementation may be associated with a reduction in the rate of hypoglycemia during admission of diabetes patients with hypoalbuminemia. 

## Figures and Tables

**Table 1:** Patients parameters across hypoglycemia groups.

		Hypoglycemia		
	All	No *n* = 159	Yes *n* = 59	*p* Value
Age (years)	77.4 ± 12.0	76.6 ± 12.3	79.6 ± 11.1	0.111
Female sex (%)	63.3	65.4	57.6	0.290
Reason for admission				
Acute infection (%)	44.5	40.3	55.9	0.038
Co-morbidities				
Hyperlipidemia (%)	54.6	51.6	62.7	0.142
Hypertension (%)	59.6	56.6	57.8	0.135
Ischemic heart disease (%)	34.9	31.4	44.4	0.082
Chronic renal failure (%)	29.4	29.6	28.8	0.914
Cerebrovascular disease (%)	9.6	10.7	6.8	0.384
Malignancy (%)	4.6	3.1	8.5	0.095
Glucose lowering medications	68.8	67.3	72.9	0.429
Insulin-all types (%)	38.5	32.7	54.2	0.004
Metformin (%)	26.6	30.8	15.3	0.021
Other (%)	18.3	18.9	16.9	0.745
Laboratory data				
Albumin during inclusion (g/dL)	3.1 ± 0.3	3.2 ± 0.3	3.1 ± 0.3	0.046
Albumin before discharge (g/dL)	3.2 ± 0.5	3.2 ± 0.5	3.0 ± 0.4	0.009
Increased albumin (%)	37.6	37.9	36.4	0.851
Average glucose (mg/dL)	174 ± 47	176 ± 49	170 ± 43	0.440
First glucose * (mg/dL)	213 ± 116	217 ± 117	200 ± 113	0.312
White blood cells (per mm^3^ × 10^3^)	11.8 ± 5.4	11.7 ± 5.1	11.9 ± 6.3	0.755
Hemoglobin (g/dL)	10.9 ± 2.1	11.1 ± 2.1	10.2 ± 2.0	0.004
C-Reactive protein (mg/dL)	8.7 ± 8.9	8.4 ± 8.8	9.5 ± 9.1	0.395
Creatinine (mg/dL)	1.9 ± 1.5	1.8 ± 1.6	2.0 ± 1.4	0.640
Total cholesterol (mg/dL)	139 ± 46	142 ± 45	132 ± 49	0.164
ALT (IU/L)	26.3 ± 41.2	27 ± 47	25 ± 22	0.726

* First glucose—the first glucose measurement during the ER admission.
